# Conversion of levoglucosan into glucose by the coordination of four enzymes through oxidation, elimination, hydration, and reduction

**DOI:** 10.1038/s41598-020-77133-8

**Published:** 2020-11-18

**Authors:** Yuya Kuritani, Kohei Sato, Hideo Dohra, Seiichiro Umemura, Motomitsu Kitaoka, Shinya Fushinobu, Nobuyuki Yoshida

**Affiliations:** 1 Department of Engineering, Graduate School of Integrated Science and Technology, Shizuoka University, 3-5-1 Johoku, Naka-ku, Hamamatsu, 432-8561 Japan; 2 Department of Science, Graduate School of Integrated Science and Technology, Shizuoka University, 836 Ohya, Suruga-ku, Shizuoka, 422-8529 Japan; 3 Research Institute of Green Science and Technology, Shizuoka University, 836 Ohya, Suruga-ku, Shizuoka, 422-8529 Japan; 4 Nihon Shokuhin Kako Co., Ltd., 30 Tajima, Fuji, 417-8530 Japan; 5 Faculty of Agriculture, Niigata University, 8050 Ikarashi 2-no-cho, Niigata, 950-2181 Japan; 6 Department of Biotechnology, The University of Tokyo, 1-1-1 Yayoi, Bunkyo-ku, Tokyo, 113-8657 Japan; 7 Collaborative Research Institute for Innovative Microbiology, The University of Tokyo, 1-1-1 Yayoi, Bunkyo-ku, Tokyo, 113-8657 Japan

**Keywords:** Multienzyme complexes, Industrial microbiology

## Abstract

Levoglucosan (LG) is an anhydrosugar produced through glucan pyrolysis and is widely found in nature. We previously isolated an LG-utilizing thermophile, *Bacillus smithii* S-2701M, and suggested that this bacterium may have a metabolic pathway from LG to glucose, initiated by LG dehydrogenase (LGDH). Here, we completely elucidated the metabolic pathway of LG involving three novel enzymes in addition to LGDH. In the S-2701M genome, three genes expected to be involved in the LG metabolism were found in the vicinity of the LGDH gene locus. These four genes including LGDH gene (*lgdA, lgdB1, lgdB2,* and *lgdC*) were expressed in *Escherichia coli* and purified to obtain functional recombinant proteins. Thin layer chromatography analyses of the reactions with the combination of the four enzymes elucidated the following metabolic pathway: LgdA (LGDH) catalyzes 3-dehydrogenation of LG to produce 3-keto-LG, which undergoes β-elimination of 3-keto-LG by LgdB1, followed by hydration to produce 3-keto-d-glucose by LgdB2; next, LgdC reduces 3-keto-d-glucose to glucose. This sequential reaction mechanism resembles that proposed for an enzyme belonging to glycoside hydrolase family 4, and results in the observational hydrolysis of LG into glucose with coordination of the four enzymes.

## Introduction

Monosaccharide anhydrides, such as levoglucosan (1,6-anhydro-β-d-glucopyranoside; LG), mannosan, and galactosan, are generated from the burning of cellulose and hemicellulose^1,2^. As the burning of biomass is a major source of particle matter, these anhydrosugars are expected to be indicators of air pollution^3^. It has been estimated that 90 million metric tons of anhydrosugars are produced every year, with the majority being LG (> 90%)^4^. As LG is also formed by pyrolysis of starch, food industries that process carbohydrates have the potential to produce LG as a by-product. We recently found that LG was produced during the industrial production process for novel water-soluble indigestible polysaccharide (resistant glucan) developed by our group, and isolated an LG-utilizing thermophile, *Bacillus smithii* S-2701M, which we used to construct an LG-removing bioprocess^5^.

Two types of microbial metabolisms of LG have been reported to date. The eukaryotic LG metabolic pathway, in fungi and yeasts, starts with LG phosphorylation with the cleavage of the 1,6-linkage by LG kinase to produce glucose 6-phosphate, which is then metabolized through the glycolytic pathway^6,7^. With respect to the bacterial metabolic pathway of LG, NAD-dependent LG dehydrogenase (LGDH) was found in *Arthrobacter* sp. I-552^8^. Recently, a gene encoding LGDH was found in the genome of *Pseudoarthrobacter phenanthrenivorans* based on the partial amino acid sequences of the LGDH in *Arthrobacter* sp. I-552, and the crystal structure of LGDH was determined^9^. The biochemical and structural analyses of LGDH confirmed the C3-specific oxidation of LG that produces 3-keto-LG. This finding led us to depict the possible LG metabolic pathway initiated by the LGDH reaction, followed by hydrolysis of 3-keto-LG and reduction of the hydrolysate to produce glucose^5,9^. However, no biochemical and genetic analyses have been performed to understand the reactions beyond the dehydrogenation of LG by LGDH in the speculated pathway.

Our previous study revealed that *B. smithii* S-2701M also possesses a thermophilic LGDH, and its glucose-forming activity was detected using the LGDH active fractions in an ion-exchange chromatography in a manner similar to that observed in *Arthrobacter* sp. I-552^5,10^. In the present study, we aimed to elucidate the LG metabolic pathway in S-2701M using the recombinant proteins including LGDH, which are expected to be involved in the pathway.

## Results

### *B. smithii* S-2701M genomic region is involved in LG metabolism

**Figure 1:**
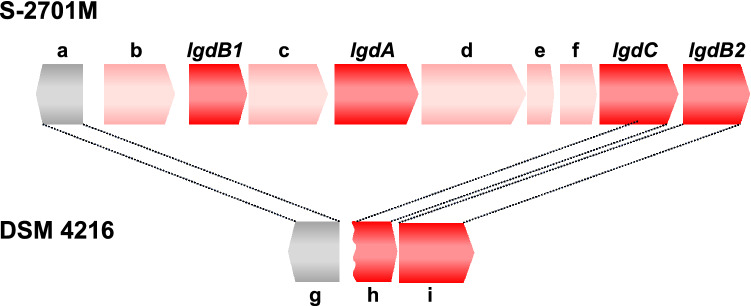
Organizations of the gene clusters involved in LG metabolism in the genomes of two *B. smithii* strains. The annotations of a-f in S-2701M genome are as follows: a, 2-haloalkanoic acid dehalogenase; b, LacI family transcriptional regulator; c, sugar ABC transporter substrate-binding protein; d, sugar ABC transporter ATP-binding protein; e, ABC transporter permease; f, ABC transporter permease. The genes g (BSM4216_1279), h (BSM4216_1280), and i (BSM4216_1281) in the DSM 4216 genome are similar to the genes a, *lgdC*, *lgdB2*, respectively, in the S-2701M genome. The DSM 4216 genome lacks regions from the gene b to 5-prime of *lgdC* in the S-2701M genome.

Based on the nucleotide and amino acid sequences of LGDH from *P. phenanthrenivorans*, the putative gene encoding LGDH was found in the genome of *B. smithii* S-2701M. Around the putative LGDH gene (*lgdA*), three genes encoding enzymes (*lgdB1*, *lgdB2*, and *lgdC*) were found (Fig. 1). The gene product of *lgdB1* was annotated as a sugar phosphate isomerase/epimerase and the closest relative was that from *Paenarthrobacter nicotinovorans* with 71% similarity (GenBank ID: GAT87749.1). The gene product of *lgdB2* was also annotated as sugar phosphate isomerase, but showed relatively lower similarity (51%) to the closest relative from *P. nicotinovorans* (GenBank ID: GAT89806.1). Intriguingly, these closest relatives were encoded by the different gene loci in the *P. nicotinovorans* genome, and there were no marked similarities between the amino acid sequences of *lgdB1* and *lgdB2*, in spite of the same annotation. The gene product of *lgdC* was annotated as a Gfo/Idh/MocA family oxidoreductase. Gfo and Idh refer to glucose-fructose oxidoreductase and inositol 2-dehydrogenase, respectively, and MocA catalyzes a dehydrogenase reaction involved in the catabolism rhizopine (3-*O*-methyl-*scyllo*-inosamine)^11^. Prior to commencing the biochemical investigation described below, we suggested that LgdC catalyzed the reduction of 3-keto-LG hydrolysate to glucose in the speculated LG metabolic pathway as described above. *lgdA*, *lgdB1*, and a part of *lgdC* were deleted in the same region in the genome of the *B. smithii* type strain DSM 4216, which is consistent with the fact that DSM 4216 could not utilize anhydrosugars such as LG and cellobiosan in our previous study^5^.

Thus, the gene products of *lgdB1*, *lgdB2*, and *lgdC* were expected to be sugar-related enzymes and involved in LG metabolism. Therefore, we attempted to express these genes and *lgdA* in *Escherichia coli* to obtain purified preparations of each protein.

### Recombinant Lgd proteins are functional in converting LG to glucose

Each *lgd* gene (*lgdA*, *lgdB1*, *lgfB2*, and *lgdC*) was expressed in *E. coli* as a GST-fusion protein, followed by removal of the GST moiety with a protease (Fig. S1). Only for the expression of *lgdB2*, *N*-lauroylsarcosine was required to obtain the gene product in a soluble fraction. Sodium dodecyl sulfate-polyacrylamide gel electrophoresis (SDS-PAGE) indicated that the estimated molecular masses of LgdA, LgdB1, LgdB2, and LgdC were 44, 30, 36, and 43 kDa, respectively, which were consistent with the molecular weight expected from the corresponding nucleotide sequences.

**Figure 2:**
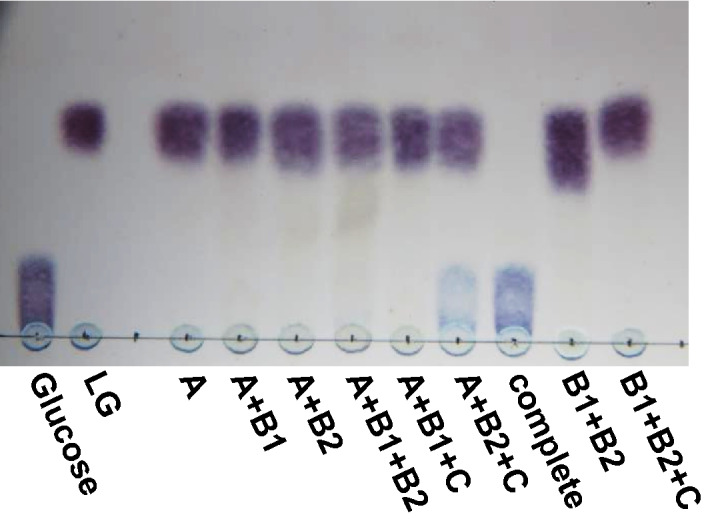
Enzyme reactions with Lgd proteins using LG as the substrate. The reaction mixture containing 50 mM HEPES-NaOH (pH 7.5), 0.1% LG, 1 mM β-NAD^+^, 1 mM MnCl_2_·4H_2_O, 10 mM 2-ME, and appropriate amount of the recombinant protein(s) were incubated at 30 °C for 15 h. Ten microliters of the reaction mixture were used for TLC analysis. A, B1, B2, and C in the figure represent LgdA, LgdB1, LgdB2, and LgdC, respectively.

Our preliminary examination indicated that LG was converted to glucose by the crude extract of S-2701M grown on LG with NAD^+^, Mn_2_^+^, and 2-mercaptoethanol (2-ME) as cofactors. Based on the reaction conditions with the crude extract, the reaction was conducted with the four recombinant Lgd proteins in various combinations using LG as the substrate with above cofactors, followed by thin layer chromatography (TLC) analysis. LG was completely converted to glucose with all four Lgd proteins (Fig. 2). However, lower level of the conversion could still be observed even without LgdB1, suggesting that LgdB2 was a partially bifunctional enzyme that could replace the function of LgdB1. NAD^+^ and Mn^2+^ were necessary in the reaction mixture for glucose formation, whereas no difference was observed with or without 2-ME (data not shown).

### LgdA is confirmed to be LGDH

The purified LgdA had a NAD-dependent dehydrogenase activity against LG (1.32 U/mg), whereas a higher activity (6.0 U/mg) was observed using 3-keto-LG, which is the reaction product obtained after LG oxidation, and NADH as the substrates. These enzyme activities were calculated by initial velocity of the change of absorbance at 340 nm due to NADH. Next, we examined the effect of the other Lgd proteins on the LGDH activity of LgdA. As shown in Table 1, the addition of LgdB1 or LgdB2 increased the specific activity (calculated by initial velocity) of LgdA. Intriguingly, these additional effects were notable when the absorbance of the reaction mixture was measured after a long time (20 min), indicating that the enzyme reaction with LgdA alone has a lower saturation point that could be prolonged by the addition of LgdB1 or LgdB2 with a consequently higher maximum saturation velocity. These results suggest that the conversion of 3-keto-LG to another compound alleviates the reaction product inhibition on LGDH.

**Table 1:** Effect of addition of the other Lgd proteins on the LGDH activity.

Additional enzyme(s)	Specific activity (unit/mg-protein)	*A*_340_
		After 20 min
None	0.44 ± 0.033	0.04 ± 0.004
LgdB1	0.98 ± 0.002	0.37 ± 0.002
LgdB2	0.52 ± 0.008	0.23 ± 0.004
LgdC	0.35 ± 0.037	0.04 ± 0.003
LgdB1, LgdB2	0.86 ± 0.004	0.37 ± 0.005
LgdB1, LgdC	0.94 ± 0.003	0.36 ± 0.004
LgdB2, LgdC	0.45 ± 0.010	0.05 ± 0.004
LgdB1, LgdB2, LgdC	0.68 ± 0.017	0.04 ± 0.004

All examinations were carried out in triplicate and the values are represented in the form of mean ± standard deviation.

### β-Elimination of 3-keto-LG is catalyzed by a novel enzyme, LgdB1

**Figure 3:**
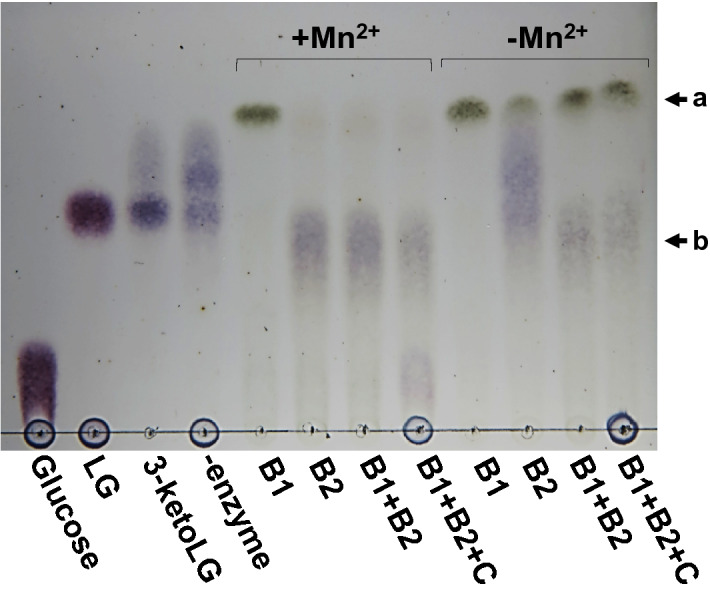
Enzyme reactions with Lgd proteins using 3-keto-LG as the substrate. The reaction mixture containing 50 mM HEPES-NaOH (pH 7.5), 0.3% 3-keto-LG, 1 mM β-NAD^+^, 1 mM MnCl_2_·4H_2_O, and appropriate amount of the recombinant protein(s) were incubated at 30 °C for 30 min. β-NADH was added to the mixture containing LgdC as one of the enzymes. Ten microliters of the reaction mixture were used for TLC analysis. B1, B2, and C in the figure represent LgdB1, LgdB2, and LgdC, respectively. The dark green and the violet spots indicated by the arrow a and b correspond to the reaction product of LgdB1 and LgdB2, respectively.

Subsequently, the reactions with the Lgd proteins were examined using 3-keto-LG as the substrate. First, we observed a new spot on TLC when only LgdB1 and 3-keto-LG were incubated. This dark green spot showed ultraviolet absorption and was expected to be the reaction product of LgdB1 (Fig. 3). When LgdB1 and LgdB2 were used as the enzymes, the dark green spot diminished and then another new violet spot was formed, which was expected to be the reaction product of LgdB2. Notably, the formation of the spot by the LgdB1 reaction was not changed with or without Mn^2+^, whereas the addition of Mn^2+^ markedly enhanced the subsequent LgdB2 reaction. We identified the structure of the compound corresponding to the dark green spot as 1,5-anhydro-d-erythro-hex-1-en-3-ulose (2-hydroxy-3-keto-d-glucal) using high resolution mass spectrometry and several nuclear magnetic resonance (NMR) analyses (Table S1 and Figs. S2 and S9).

### Glucose is formed by subsequent hydration and reduction

**Figure 4:**
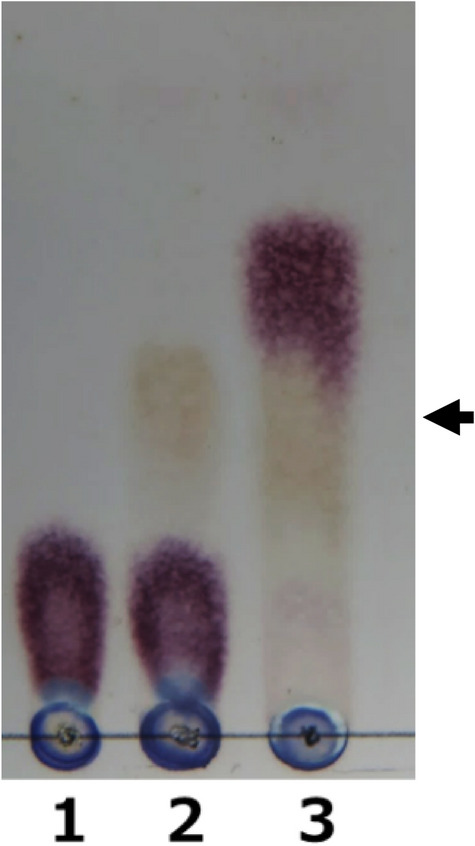
LgdC reaction using glucose as the substrate (reverse reaction). The reaction mixture for lane 1: 50 mM Tris-HCl (pH 9.0), 0.1% glucose, and 1 mM β-NAD^+^; that for lane 2: 10 μg/ml LgdC was added to the reaction mixture for lane 1; that for lane 3: 50 mM HEPES-NaOH (pH 7.5), 0.1% LG, 1 mM β-NAD^+^, 1 mM MnCl_2_·4H_2_O, and 5 mg/ml each of LgdA, LgdB1, and LgdB2. The reaction was carried out at 30 ℃ for 15 h and 10 μl of the reaction mixture was used for TLC analysis. An arrow indicates the spots corresponding to the product of the reverse reaction of LgdC and that of the forward reaction by LgdA, LgdB1, and LgdB2.

We also attempted to identify the structure of the compound expected to be the reaction product of LgdB2. However, the compound was unstable and degraded rapidly during the extraction and purification of the compound. Hence, we used another approach to identify the compound. As described above, LgdC was expected to be an oxidoreductase using NAD(P) as the cofactor. When NADH was added to the reaction mixture containing 3-keto-LG as the substrate and containing LgdB1, LgdB2, and LgdC as the enzymes, the violet spot that was considered to be the LgdB2 reaction product became weak and the spot corresponding to glucose was observed (Fig. 3). These result suggest that LgdC reduces C3-keto group of the LgdB2 reaction product to form glucose; accordingly, the reaction product of LgdB2 is presumed to be 3-keto-d-glucose. Indeed, when LgdC was reacted with glucose and NAD^+^ as the substrate and the cofactor, respectively, the violet spot showed the same migration as that corresponding to the LgdB2 reaction product (Fig. 4). Furthermore, marked reduction of NAD^+^ in the reaction mixture was also confirmed by spectrophotometry (data not shown).

**Figure 5:**
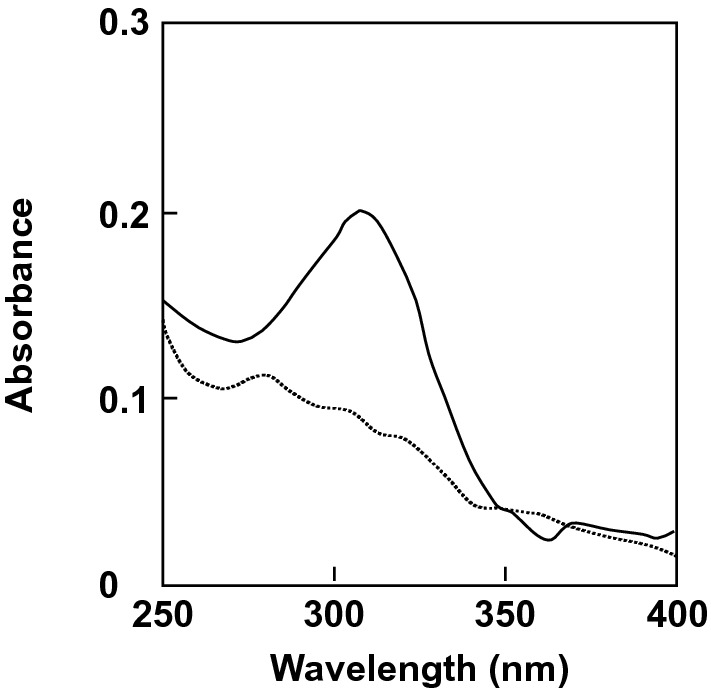
Determination of 3-keto-d-glucose by the Hayano and Fukui method. Four microliters of the reaction product of LgdB2 extracted with water from a TLC plate was mixed with 1 μl of 1 M potassium phosphate buffer (pH 7.0) and incubated at room temperature for 3 min. Solid line, the absorption spectrum in the buffer; dotted line, the absorption spectrum in water.

In 1969, Fukui and Hayano reported the method to determine 3-keto-sucrose and 3-keto-d-glucose^12^. They showed that an absorption maximum at 310 nm was observed when 3-keto-d-glucose was incubated in potassium phosphate buffer (pH 7.0). In this study, the violet spot expected to be the LgdB2 reaction product was extracted from a TLC plate and immediately incubated with potassium phosphate buffer. Consequently, an absorption maximum of the solution was observed at 310 nm as shown in Fig. 5. Furthermore, mass spectrum analysis showed that the LgdB2 reaction product extracted from the TLC spot had a *m/z* of 379.08 (calculated for C_12_H_20_O_12_ [2M + Na^+^]), suggesting a keto-glucose (Fig. S10). Taken together, these findings suggest that the LgdB2 reaction product is 3-keto-d-glucose.

Accordingly, we speculate that LgdB2 catalyzes the hydration of the 1,2-double bond of 2-hydroxy-3-keto-d-glucal to produce 3-keto-d-glucose, and LgdC is glucose-3-dehydrogenase (G3DH), through which 3-keto-d-glucose is reduced to glucose.

## Discussion

**Figure 6:**
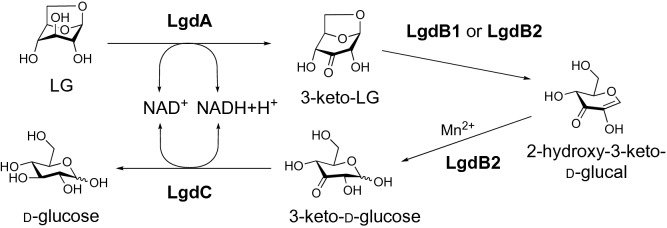
Proposed LG metabolic pathway in *B. smithii* S-2701M.

In this study, we completely elucidated the metabolism of LG to glucose and showed that it involved four enzymes, namely, LgdA, LgdB1, LgdB2, and LgdC. As shown in Fig. 6, our proposed pathway involves C3-oxidation by LgdA (LGDH), cleavage of the C-O bond of the 1,6-linkage through β-elimination catalyzed by LgdB1, and hydration of the 2-hydroxy-3-keto-d-glucal to produce 3-keto-d-glucose by LgdB2, followed by reduction to glucose by LgdC. This reaction mechanism resembles those proposed for the enzymes belonging to the glycosyl hydrolase family 4 (GH4)^13–17^. Prior to elucidating the LG metabolic pathway, we had expected similar reaction mechanisms between LG degradation and GH4-type hydrolysis. Most GH4 enzyme reactions also require NAD^+^, Mn^2+^, and relatively large amounts of a reducing reagent such as dithiothreitol or 2-ME. The reaction mechanism of GH4 enzyme has been well-studied in α-galactosidase (MelA) from *Citrobacter freundii*^16^. In this mechanism, NAD^+^ requires the oxidation of the C3-hydroxy group in a galactose moiety, and Mn^2+^ is necessary for increasing polarization at the position of C3 to form an enolate anion before β-elimination. Reportedly, the reducing agent protects the oxidation of reactive Cys residue^13^. Recently, Bell et al. reported a novel GH4 type hydrolase (*Rg*NanOx) from the human gut symbiont *Ruminococcus gnavus* which converted 2,7-anhydro-*N*-acetylneuraminic acid into *N*-acetylneuraminic acid^18^. The reaction mechanism is thought to be close to that of LG conversion into glucose in S-2701M as anhydrosugars are the substrates in both reactions. Thus, in GH4 type hydrolases, a single polypeptide catalyzes four reactions, namely, dehydrogenation, β-elimination, hydration, and reduction, whereas the four enzymes examined in this study together act as a GH4-type enzyme and Mn^2+^ was required after β-elimination for the hydration of 3-keto-d-glucose in the LG degradation pathway in S-2701M. When LG was used as the substrate, the reaction product of LgdB1 was not detected on TLC, whereas the weak spots corresponding to the LgdB2 reaction product was observed when LgdA, LgdB1, and LgdB2 were used as the enzymes (Fig. 2), suggesting that LGDH reaction catalyzed by LgdA is rate-limiting in the *in-vitro* reconstitution reaction using LG (6 mM) as the substrate. As described above, the LGDH activity of LgdA in reverse reaction (reduction of 3-keto-LG) was higher than that in forward reaction (dehydrogenation of LG), which was consistent with our previous result in LGDH of *P. phenanthrenivorans*^9^. However, the addition of LgdC caused the marked degradation of LG (Fig. 2), which may be due to NAD^+^ regeneration accompanying glucose formation by LgdC reaction. Furthermore, in the presence of Mn^2+^ ion, the second violet spot expected to correspond to 3-keto-d-glucose was observed from 3-keto-LG using only LgdB2 at the same level as that when LgdB1 and LgdB2 were used (Fig. 3), suggesting that LgdB2 is a bifunctional enzyme that catalyzes the β-elimination of 3-keto-LG with low affinity and hydration of 2-hydroxy-3-keto-d-glucal. This suggestion is also consistent with the results using LG as the substrate and it seemed that glucose formation was weak only when LgdB2 was used, which was probably due to a low concentration of 3-keto-LG (Fig. 2). The additional effect of LgdB1 and LgdB2 on LgdA activity (Table 1) also supported the bifunctional characteristic of LgdB2.

Only a few enzymes capable of cleaving glycosyl linkages via β-elimination have been reported; these are known as polysaccharide lyases and include pectin lyase^19^, alginate lyase^20^, and glucuronan lyase^21^. Polysaccharide lyases catalyze the cleavage of a glycoside linkage of each polysaccharide from the non-reducing end in a β-elimination manner. The LgdB1 catalyzes an intramolecular glucoside linkage in LG; thus, LgdB1 is thought to be a novel enzyme, which can be called 3-keto-LG decyclase or 2-hydroxy-3-keto-d-glucal synthase (EC 5.5). LgdB2 also has a unique characteristic and catalyzed the hydration of 2-hydroxy-3-keto-d-glucal, and hence, it can be called 3-keto-d-glucose-1,2-dehydratase. It is known that several sugar dehydratases catalyze 4,6-hydration of hexoses to form 6-deoxysugars, and are members of the short chain dehydrogenase/reductase protein superfamily^22–24^. The reaction of the sugar 4,6-dehydratases also involves β-elimination in the 4,6-dehydration to eliminate the C6-hydroxyl group, whereas the dehydration in the LG metabolic pathway occurs in a compound after β-elimination.

We speculate that LgdC catalyzes the oxidation of C3 position of glucose. Only a few enzymes oxidize a hydroxyl group at C3 position in sugars, and G3DHs have been found in *Cytophaga marinoflava*^25^, *Halomonas* sp.^26^, and *Agaricus bisporus*^27^. It is known that these G3DHs cannot use NAD^+^ as the cofactor, but can use artificial cofactors such as 2,6-dichloroindophenol. LgdC was also thought to be G3DH and could use NAD^+^ as the cofactor in this study. LGDH can also oxidize the C3-hydroxyl group of LG, which is the key reaction in the LG metabolic pathway. However, its activity toward d-glucose is very low; 0.05% and 5% activities of LG have been found in *Pseudoarthrobacter* and *Arthrobacter* LGDHs, respectively^8,9^, whereas no detectable activity toward d-glucose was observed in S-2701M LGDH in the current study (data not shown). Thus, LgdC catalyzing C3-oxidation of glucose is also thought to be a novel enzyme. Simple hydrolytic enzyme on the β-1,6-linkage of LG has not been reported so far, suggesting the difficulty in cleaving the linkage through the common mechanism of glycoside hydrolases. We reported that the oxidation of LG at C3 caused the linkage to be unstable under neutral-alkaline conditions^28^. The complex pathway reported here through oxidation, β-elimination, hydration and reduction may utilize the instability, and results in just the intramolecular hydrolysis of the linkage that is difficult to cleave.

In conclusion, this study revealed that four enzymes, including three novel ones coordinated the conversion of LG to glucose in *B. smithii* S-2701M. LG is known to be produced by pyrolysis or supercritical water treatment of lignocellulosic biomass^29,30^. Typically, saccharification of lignocellulose is a bottleneck for its utilization as feedstock for subsequent fermentation process. Recently, researchers have paid considerable attention to microbial conversion of LG as a biomass-derived compound^31–33^. Thus, effective utilization of LG would lead to a novel and an effective utilization of cellulosic biomass. However, only fungal LG kinases have been used to construct the recombinant fermentation systems. It is expected that the bacterial LG metabolism involving the *lgd* genes will be applied in the recombinant fermentation system for LG. Alternatively, S-2701M could utilize LG at the same level as glucose, which is free from catabolite repression^5^, suggesting that this bacterium can be an effective host for production of useful compounds from LG. There are reports indicating another application of LG as a useful starting material, and that the ring-opening polymerization of 1,6-anhydrohexopyranose can be used for the synthesis of stereoregular polysaccharides with biological activities, such as anti-HIV reagents and artificial biocompatible materials^34–36^. The reaction mechanism of LG degradation elucidated in the present study may also contribute to the synthesis of such anhydrosugars.

## Methods

### Materials

LG was purchased from MSD K.K., Tokyo, Japan. 3-keto-LG was enzymatically synthesized as described previously^27^.

### Expression of Lgd proteins in *Escherichia coli*

The region involved in LG metabolism was searched in the draft genome of *B. smithii* S-2701M (accession no. LC529899) according to the amino acid sequence of LGDH from *P. phenanthrenivorans*^9^. *lgd* genes in this region were amplified from the S-2701M genome by PCR using primer sets with 5′-BamHI and 3′-SalI restriction sites for *lgdA*, *lgdB1*, and *lgdB2*, and 5′-EcoRI and 3′-SalI restriction sites for *lgdC*, respectively. The primers used in this study are listed in Table S2. Each PCR fragment was introduced into pGEX-6P-1 digested with appropriate restriction enzymes. *E. coli* DH5α [F^−^ *Φ*80*lacZ*ΔM15 Δ(*lacZYA*-*argF*)U169 *deoR recA*1 *hsdR*17(r_K_^−^ m_K_^+^) *phoA supE*44 *λ*^−^ *thi*^−1^ *gyrA*96 *relA*1] was used for expression of each plasmid.

The recombinant *E. coli* was cultivated at 30°C in LB medium containing 50 μg/ml ampicillin, and isopropyl-β-d-thiogalactopyranoside (0.1 mM) was added to the medium when OD_660_ reached 0.5. The overnight culture was centrifuged, washed with 0.85% KCl, and suspended with PBS buffer (140 mM NaCl, 2.7 mM KCl, 10 mM Na_2_HPO4, 1.8 mM KH_2_PO4, 1 mM dl-dithiothreitol (DTT), pH 7.3). For purification of LgdB2, *N*-lauroylsarcosine was added to the PBS buffer. The cells were disrupted with zirconia beads and the cell extract was obtained by centrifugation at 20,400 × *g* for 10 min. Each recombinant protein fused with a glutathione *S*-transferase was recovered from the cell extract by using a Glutathione Sepharose 4B resin (GE Helthcare Japan, Tokyo). The resin was transferred to a column and washed with the cleavage buffer (50 mM Tris-HCl, 150 mM NaCl, 1 mM DTT, pH 7.5). On-column cleavage of a GST moiety was carried out with a PreScission Protease (GE Healthcare Japan) and the elution was further used as the purified enzyme preparation.

### Method for the enzyme reactions

The reaction was carried out with a combination of the recombinant enzymes using LG or 3-keto-LG as the substrate, followed by TLC analysis. The reaction mixture using LG as the substrate contained 50 mM HEPES-NaOH, 1% LG, 1 mM β-NAD^+^, 1 mM MnCl_2_·4H_2_0, 10 mM 2-ME, and appropriate amount of the recombinant protein(s) (pH 7.5). When 3-keto-LG was used as the substrate, β-NAD^+^ was removed from the reaction mixture, and β-NADH was added to the mixture containing LgdC as one of the enzymes. After reaction for 30 min-overnight (30°C), 10 μl of the reaction mixture was spotted onto a TLC plate (TLC Silica Gel 60 F254, Merck Japan, Tokyo). The plate was developed with acetonitrile/water (9:1, *v/v*) and sugar-related compounds were detected with 2% 1,3-dihydroxynaphthalene in 95% ethanol.

The activities of LGDH and G3DH were measured spectrophotometrically at 25 °C by following the increasing absorbance at 340 nm in the reaction mixture containing 50 mM Tris-HCl, 1 mM β-NAD^+^, and appropriate of the enzyme solution. No difference was observed in each enzyme assay with the other kinds of buffer (phosphate and HEPES).

### Identification of reaction products

The reaction product of LgdB1 was obtained from LG with LgdA and LgdB1 and NAD regenerative reaction with lactate dehydrogenase (LDH) was used to enhance the LgdA reaction. The reaction was carried out at 30 °C for 17 h in the reaction mixture consisting of 50 mM HEPES-NaOH (pH 7.5), 1% LG, 1 mM β-NAD^+^, 31 mM sodium pyruvate, 1 U/ml LDH (from pig heart, TOYOBO Co., Ltd., Osaka), 15 μg/ml LgdA, and 20 μg/ml LgdB1. Total 24 ml of the reaction mixture was subjected to TLC analysis as described above. High-resolution mass spectrometry was conducted on a Bruker Compact (ESI-Q-TOF). Nuclear magnetic resonance (NMR) spectra were recorded on a Bruker AVANCE III HD with CryoProbe Prodigy (^1^H 400 MHz, ^13^C 100 MHz) spectrometer in D_2_O–H_2_O (10:90) at 298 K. Chemical shifts for ^1^H NMR are expressed in parts per million (ppm) relative to MeOH (*δ* 3.34 ppm). Chemical shifts for ^13^C NMR are expressed in ppm relative to MeOH (*δ* 49.5 ppm).

The reaction product of LgdB2 was determined according to the method of Fukui and Hayano^12^. The reaction mixture containing 30 mM HEPES-NaOH (pH 7.0), 0.3% 3-keto-LG, 1 mM MnCl_2_·4H_2_0, and 15 μg/ml LgdB2 was incubated at 30 °C for 30 min. The reaction mixture (40 μl) was subjected to TLC analysis as described above.

The reaction product was extracted at 4  °C for 10 min from the corresponding spot with 60 μl of distilled water. Four microliters of the extract were mix with 1 μl of 1 M potassium phosphate buffer (pH 7.0) and the absorption spectrum was measured after 3 min.

## Supplementary information

Supplementary Information.

## References

[1] Simoneit BRT, Schauer JJ, Nolte CG, Oros DR, Elias VO, Fraser MP, Rogge WF, Cass GR (1999). Levoglucosan, a tracer for cellulose in biomass burning and atmospheric particles. Atmos. Environ..

[2] Nolte CG, Schauer JJ, Cass GR, Simoneit BRT (2001). Highly polar organic compounds present in wood smoke and in the ambient atmosphere. Environ. Sci. Technol..

[3] Janoszka K, Czaplicka M (2019). Methods for the determination of levoglucosan and other sugar anhydrides as biomass burning tracers in environmental samples—A review. J. Sep. Sci..

[4] Lian J, Choi J, Tan YS, Howe A, Wen Z, Jarboe LR (2016). Identification of soil microbes capable of utilizing cellobiosan. PLoS ONE.

[5] Iwazaki S, Hirai H, Hamaguchi N, Yoshida N (2018). Isolation of levoglucosan-utilizing thermophilic bacteria. Sci. Rep..

[6] Kitamura Y, Yasui T (1991). Purification and some properties of levoglucosan (1,6-anhydro-β-d-glucopyranose) kinase from the yeast *Sporobolomyces salmonicolor*. Agric. Biol. Chem..

[7] Rother C, Gutmann A, Gudiminchi R, Weber H, Lepak A, Nidetzky B (2018). Biochemical characterization and mechanistic analysis of the levoglucosan kinase from *Lipomyces starkeyi*. ChemBioChem.

[8] Nakahara K, Kitamura Y, Yamagishi Y, Shoun H, Yasui T (1994). Levoglucosan dehydrogenase involved in the assimilation of levoglucosan in *Arthrobacter* sp. I-552. Biosci. Biochem. Biotechnol..

[9] Sugiura M (2018). Identification, functional characterization, and crystal structure determination of bacterial levoglucosan dehydrogenase. J. Biol. Chem..

[10] Yasui T, Kitamura Y, Nakahara K, Abe Y (1991). Metabolism of levoglucosan (1,6-anhydro-β-d-glucopyranose) in bacteria. Agric. Biol. Chem..

[11] Taberman H, Parkkinen T, Rouvinen J (2016). Structural and functional features of the NAD(P) dependent Gfo/Idh/MocA protein family oxidoreductases. Protein Sci..

[12] Fukui S, Hayano K (1969). Micro methods for determination of 3-ketosucrose and 3-ketoglucose. Agric. Biol. Chem..

[13] Lodge JA, Maier T, Liebl W, Hoffmann V, Sträter N (2003). Crystal structure of *Thermotoga maritima* α-glucosidase AglA defines a new clan of NAD^+^-dependent glycosidases. J. Biol. Chem..

[14] Yip VL, Withers SG (2006). Mechanistic analysis of the unusual redox-elimination sequence employed by *Thermotoga maritima* BglT: A 6-phospho-β-glucosidase from glycoside hydrolase family 4. Biochemistry.

[15] Huang W, Llano J, Gauld JW (2010). Redox mechanism of glycosidic bond hydrolysis catalyzed by 6-phospho-α-glucosidase: A DFT study. J. Phys. Chem..

[16] Chakladar S, Cheng L, Choi M, Liu J, Bennet AJ (2011). Mechanistic evaluation of MelA α-galactosidase from *Citrobacter freundii*: A family 4 glycosyl hydrolase in which oxidation is rate-limiting. Biochemisty.

[17] Yun BY, Jun SY, Kim NA, Yoon BY, Piao S, Park SH, Jeong SH, Lee H, Ha NC (2011). Crystal structure and thermostability of a putative α-glucosidase from *Thermotoga neapolitana*. Biochem. Biopsy’s. Res. Commun..

[18] Bell A (2020). Uncovering a novel molecular mechanism for scavenging sialic acids in bacteria. J. Biol. Chem..

[19] Phaff HJ, Demain AL (1956). The unienzymatic nature of yeast polygalacturonase. J. Biol. Chem..

[20] Davidson IW, Sutherland IW, Lawson CJ (1976). Purification and properties of an alginate lyase from a marine bacterium. Biochem. J..

[21] Konno N, Igarashi K, Habu N, Samejima M, Isogai S (2009). Cloning of the *Trichoderma reesei* cDNA encoding a glucuronan lyase belonging to a novel polysaccharide lyase family. Appl. Environ. Microbiol..

[22] Allard STM, Giraud MF, Whitfield C, Graninger M, Messner P, Naismith JH (2001). The crystal structure of dTDP-d-glucose 4,6-dehydratase (RmlB) from *Salmonella enterica* serovar Typhimurium, the second enzyme in the dTDP-l-rhamnose pathway. J. Mol. Biol..

[23] Koropatkin NM, Holden HM (2005). Structure of CDP-d-glucose 4,6-dehydratase from *Salmonella typhi* complexed with CDP-d-xylose. Acta Crystallogr. Sect. D Biol. Crystallogr..

[24] Pfeiffer M, Johansson C, Krojer T, Kavanagh KL, Oppermann U, Nidetzky B (2019). A parsimonious mechanism of sugar dehydration by human GDP-mannose-4,6-dehydratase. ACS Catal..

[25] Tsugawa S, Horiuchi S, Tanaka M, Wake SK (1996). Purification of a marine bacterial glucose dehydrogenase from *Cytophaga marinoflava* and its application for measurement of 1,5-anhydro-d-glucitol. Appl. Biochem. Biotechnol..

[26] Tsugawa W, Ogasawara N, Sode K (1998). Fluorescent measurement of 1,5-anhydro-d-glucitol based on a novel marine bacterial glucose dehydrogenase. Enzyme Microbial. Technol..

[27] Morrison SC, Wood DA, Wood PM (1999). Characterization of a glucose 3-dehydrogenase from the cultivated mushroom (*Agaricus bisporus *). Appl. Microbiol. Biotechnol..

[28] Kitaoka M (2017). Synthesis of 3-keto-levoglucosan using pyranose oxidase and its spontaneous decomposition via β-elimination. J. Appl. Glycosci..

[29] Islam ZU, Zhisheng Y, Hassan EB, Dongdong C, Hongxun Z (2015). Microbial conversion of pyrolytic products to biofuels: A novel and sustainable approach toward second-generation biofuels. J. Ind. Microbiol. Biotechnol..

[30] Ehara K, Saka S (2002). A comparative study on chemical conversion of cellulose between the batch-type and flow-type systems in supercritical water. Cellulose.

[31] Layton DS, Ajjarapu A, Choi DW, Jarboe LR (2011). Engineering ethanologenic *Escherichia coli* for levoglucosan utilization. Bioresour. Technol..

[32] Kim EM, Um Y, Bott M, Woo HM (2015). Engineering of *Corynebacterium glutamicum* for growth and succinate production from levoglucosan, a pyrolytic sugar substrate. FEMS Microbiol. Lett..

[33] Xiong X, Lian J, Yu X, Garcia-Perez M, Chen S (2016). Engineering levoglucosan metabolic pathway in *Rhodococcus jostii* RHA1 for lipid production. J. Ind. Microbiol. Biotechnol..

[34] Yoshida T, Nakashima H, Yamamoto N, Uryu T (1993). Anti-AIDS virus activity *in vitro* of dextran sulfates obtained by sulfation of synthetic and natural dextrans. Polym. J..

[35] Satoh T, Imai T, Ishihara H, Maeda T, Kitajyo Y, Narumi A, Kaga H, Kaneko N, Kakuchi T (2003). Synthesis of hyperbranched polysaccharide by thermally induced cationic polymerization of 1,6-anhydro-β-d-mannopyranose. Macromolecules.

[36] Takahashi K, Satoh H, Satoh T, Kakuchi T, Miura M, Sasaki A, Sasaki M, Kaga H (2009). Formation kinetics of levoglucosan from glucose in high temperature water. Chem. Eng. J..

