# Supplementary material for: Conversion of levoglucosan into glucose by the coordination of four enzymes through oxidation, elimination, hydration, and reduction
Source: Sci Rep. 2020 Nov 18;10:20066. doi: 10.1038/s41598-020-77133-8 (PMC7676230; doi:10.1038/s41598-020-77133-8)
Supplement: Supplementary file 1 — Supplementary Information. [file 41598_2020_77133_MOESM1_ESM.docx]

**Supplementary Information**

Title: **Conversion of levoglucosan into glucose by the coordination of four enzymes through oxidation, elimination, hydration, and reduction**

Authors: Yuya Kuritani^a^, Kohei Sato^a^, Hideo Dohra^b, c^, Seiichiro Umemura^d^, Motomitsu Kitaoka^e^, Shinya Fushinobu^f, g^, and Nobuyuki Yoshida^a^*

^a^Department of Engineering, Graduate School of Integrated Science and Technology, Shizuoka University, 3-5-1 Johoku, Naka-ku, Hamamatsu 432-8561, Japan; ^b^Department of Science, Graduate School of Integrated Science and Technology, Shizuoka University, 836 Ohya, Suruga-ku, Shizuoka 422-8529, Japan; ^c^Research Institute of Green Science and Technology, Shizuoka University, Shizuoka, Japan; ^d^Nihon Shokuhin Kako Co., Ltd., 30 Tajima, Fuji 417-8530, Japan; ^e^Faculty of Agriculture, Niigata University, 8050 Ikarashi 2-no-cho, Niigata 950-2181, Japan; ^f^Department of Biotechnology and ^g^Collaborative Research Institute for Innovative Microbiology, The University of Tokyo, 1-1-1 Yayoi, Bunkyo-ku, Tokyo 113-8657, Japan.

*To whom correspondence may be addressed. Email: yoshida.nobuyuki@shizuoka.ac.jp

**This file includes:**

**•Original images of Fig. 2, 3, and 4.**

**•Fig. S1-S10.**

**•Table S1 and S2.**

**Original images of Fig. 2, 3, and 4.**

**
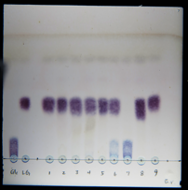

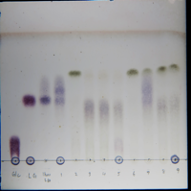
**

**Fig. 2 Fig. 3**

**
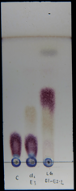
**

**Fig. 4**

**Fig. S1: Expression of *lgd* genes in *E. coli*.**

Each *lgd* gene was expressed in *E. coli* as a GST-fused protein as described in “Materials and methods”. After separation of each GST-fused protein by a Glutathione Sepharose 4B resin, on-column protease digestion was carried out to obtain each purified Lgd protein. M, molecular mass marker; 1, crude extract; 2, flow through fraction; 3, after protease digestion.

**Table S1 ^1^H and ^13^C NMR and HMBC data of 2-hydroxy-3-keto-d-glucal.**

| Position | *δ*_C_*^a,b^* | *δ*_H_*^a^* (*J* in Hz) | HMBC |
| --- | --- | --- | --- |
| 1 | 151.5, CH | 7.53, s |  |
| 2 | 133.6, C |  | H-1 |
| 3 | 193.0, C |  | H-1, H-4 |
| 4 | 68.0, CH | 4.53, d (13.4) | H-6, H-6’ |
| 5 | 83.2, CH | 4.24, ddd (13.4, 4.6, 1.9) | H-1, H-4, H-6 |
| 6 | 60.6, CH_2_ | 3.98, dd (12.9, 4.6) | H-4 |
| 6’ |  | 3.90, dd (12.9, 1.9) |  |
| *^a^*The chemical shifts are in *δ* value (ppm) from MeOH.  *^b^*Multiplicities were assigned by DEPT spectra. | | | |

**Table S2 PCR primers used in this study.**

| Target gene | | Sequence (5´→3´) |
| --- | --- | --- |
| *lgdA* (forward) | AAT GGA TCC AAA AAA TTG AAT GTA GGT ATG ATT GG | |
| (reverse) | ATT GTC GAC CTA TTT TAA TTC CGC CG | |
| *lgdB1* (forward) | AAT GGA TCC AAA TTC GGT TAT CAA ACG AAT ACA | |
| (reverse) | ATT GTC GAC TCA AAC TTG ATT CGT TTT TTC AAA | |
| *lgdB2* (forward) | AAT GGA TCC AAA CTA GGC GTA TTT ACC GTT CTT TAT | |
| (reverse) | ATT GTC GAC TTA AGC CCA CCA CAT TTC TTC TAC C | |
| *lgdC* (forward) | AAA TGA ATT CGA AAA AAT AAA AGT GGG AAT CAT CG | |
| (reverse) | ATT GTC GAC CTA ATC TGC CTT CAC TTT CAC | |

**Fig. S2: ^1^H NMR spectrum of 2-hydroxy-3-keto-d-glucal (water suppression)**
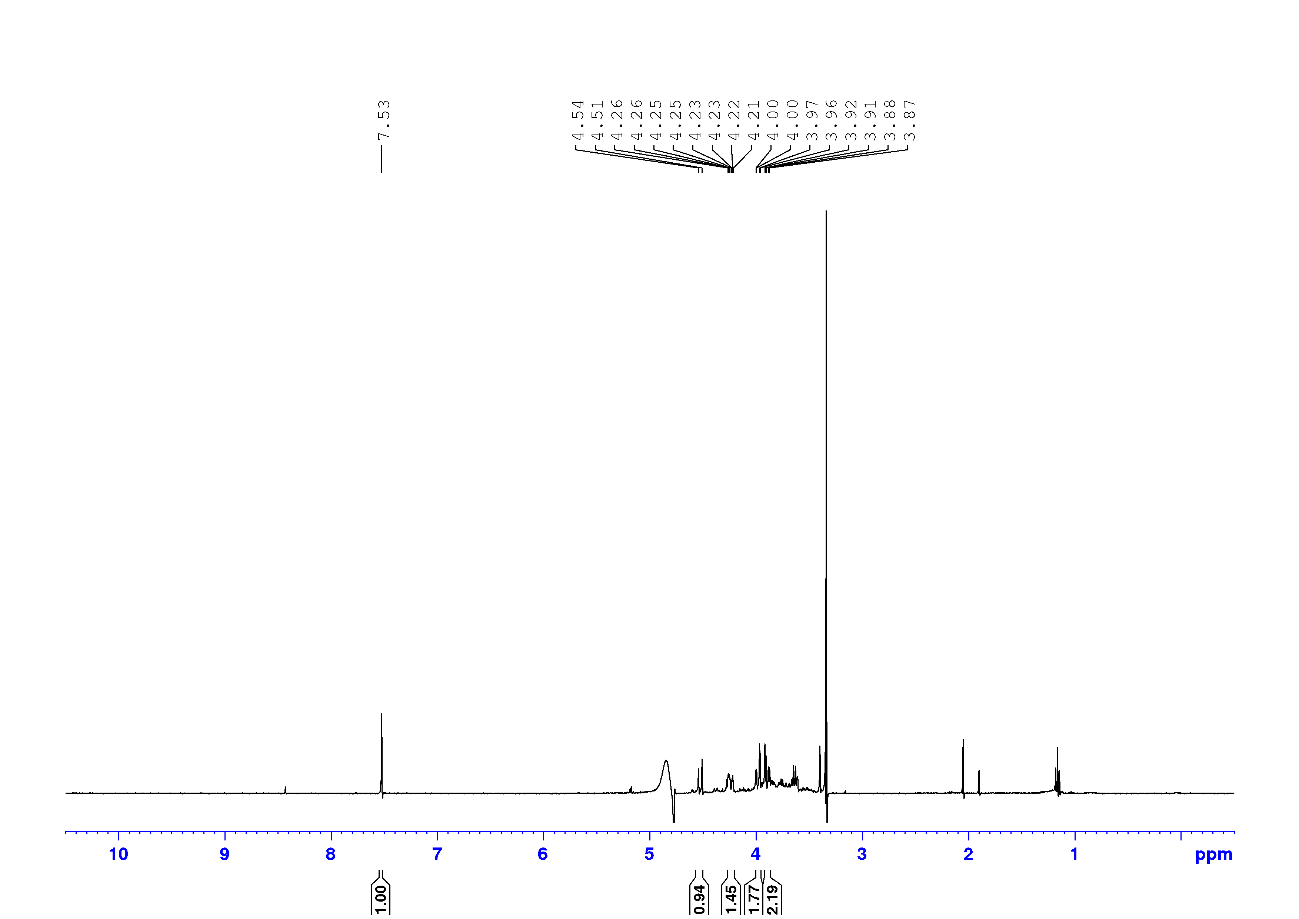
**.**

**Fig. S3: ^13^C NMR spectrum of 2-hydroxy-3-keto-d-glucal**
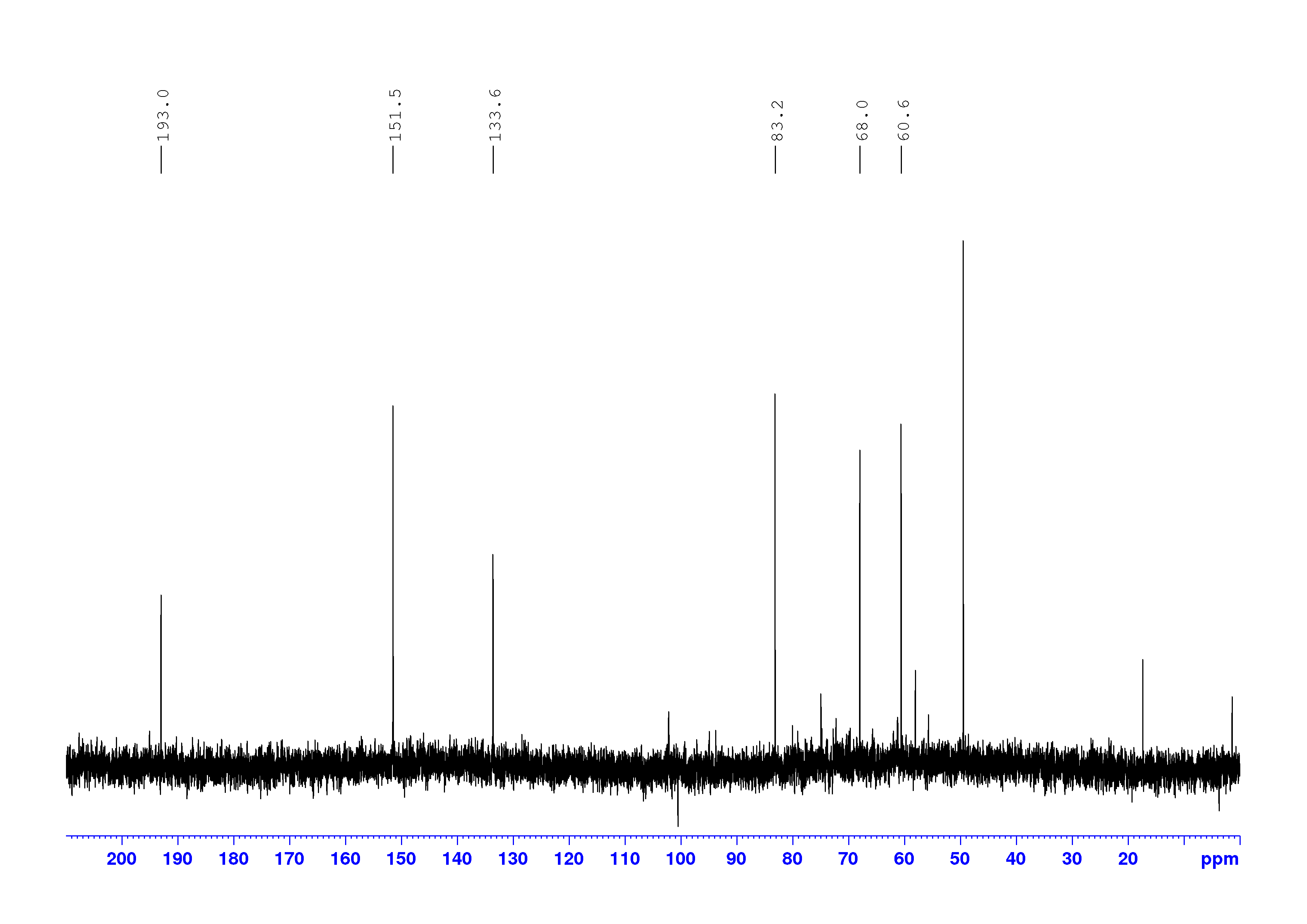
**.**

**Fig. S4: DEPT 135 spectrum of 2-hydroxy-3-keto-d-glucal.**


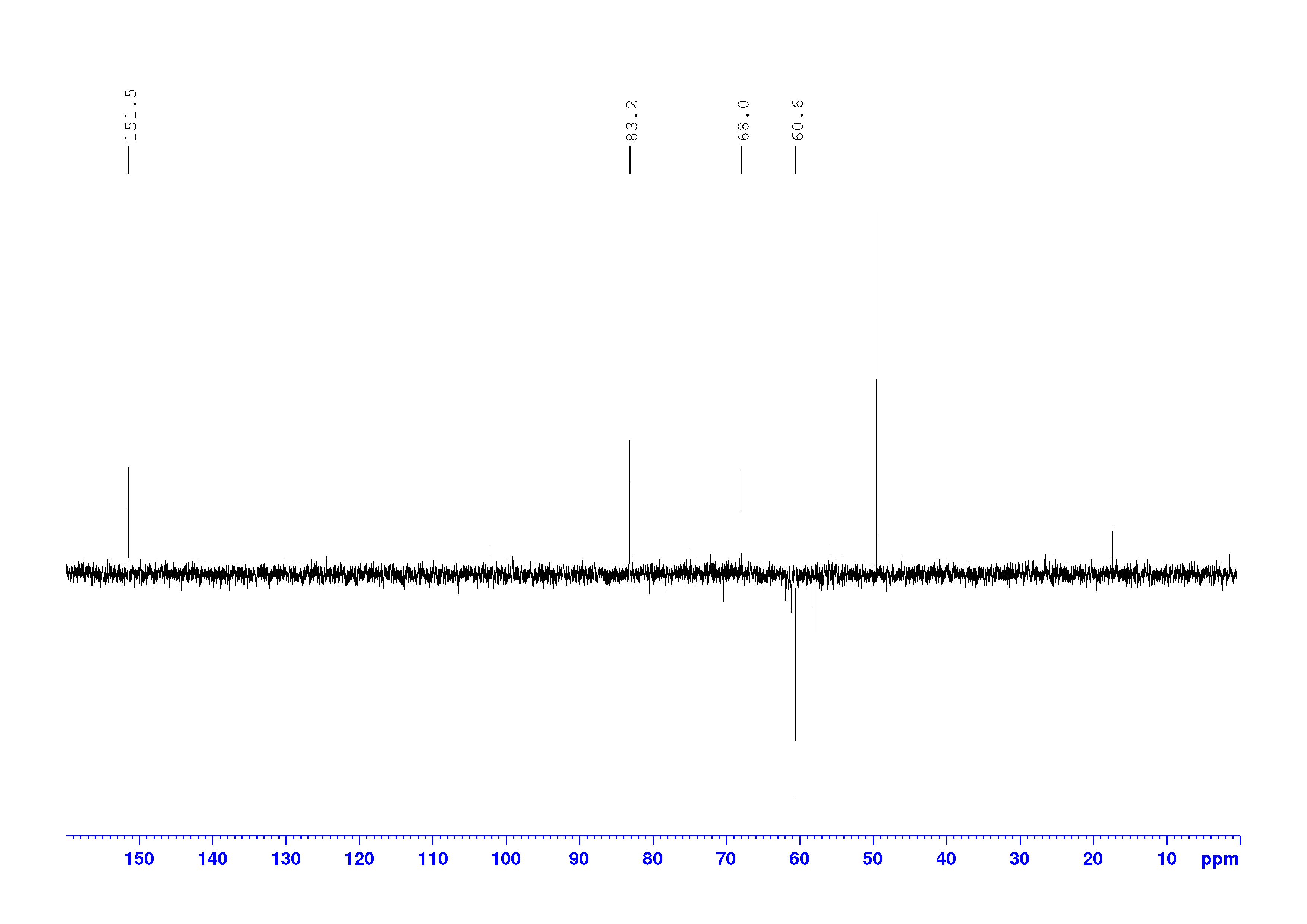


**Fig. S5: DEPT 90 spectrum of 2-hydroxy-3-keto-d-glucal**
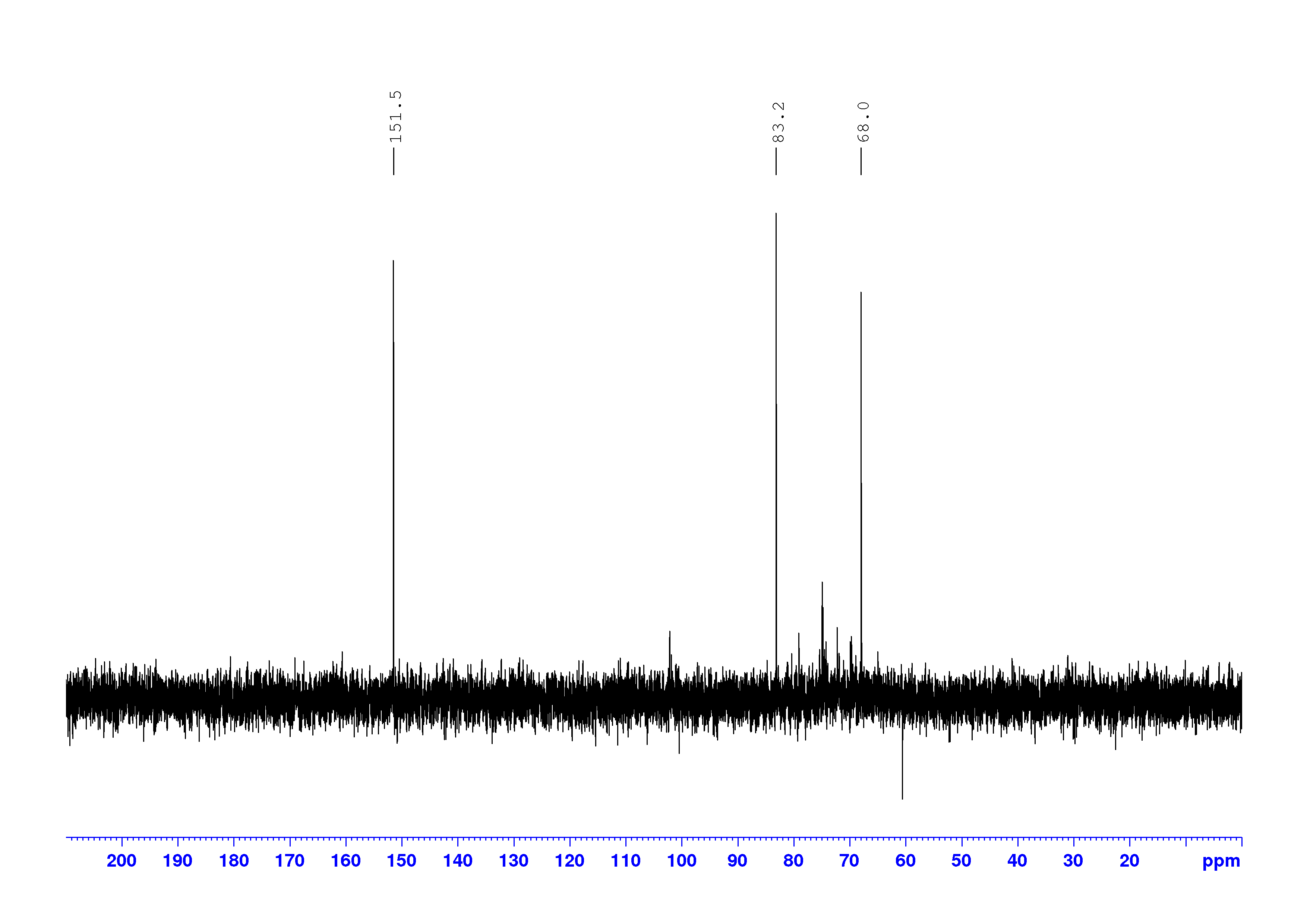
**.**

**Fig. S6: COSY spectrum of 2-hydroxy-3-keto-d-glucal**
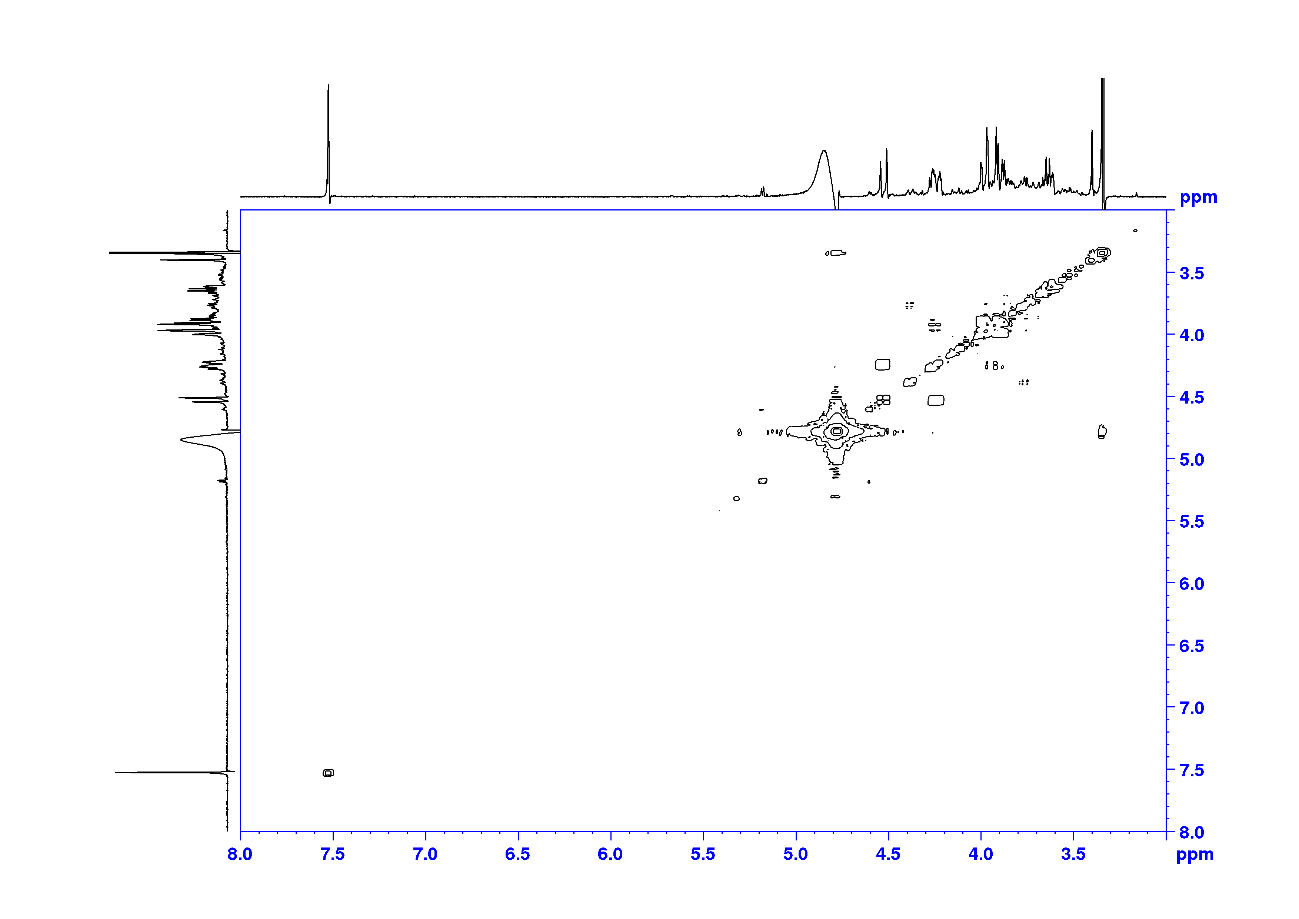
**.**

**Fig. S7: HSQC spectrum of 2-hydroxy-3-keto-d-glucal**
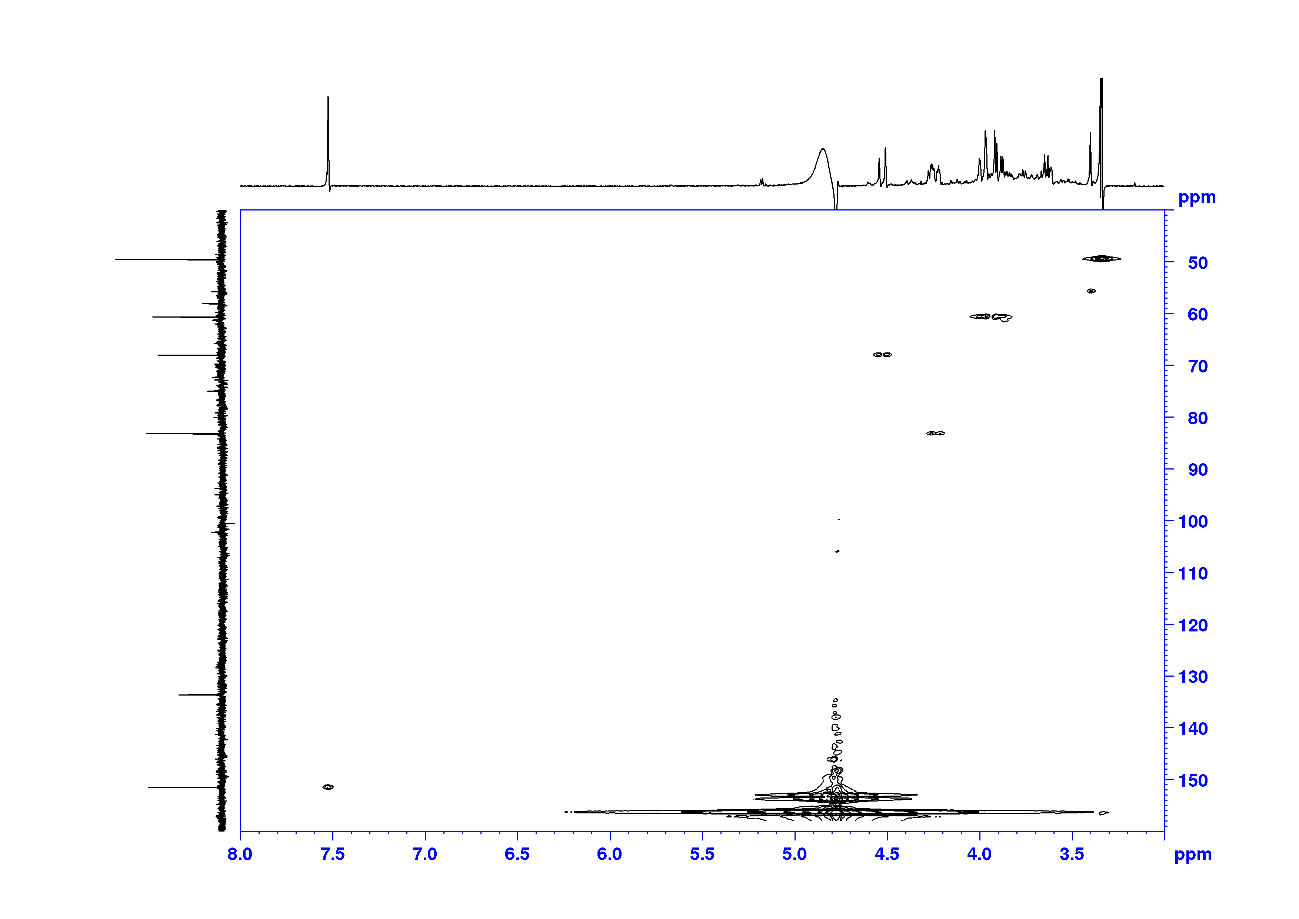
**.**

**Fig. S8: HMBC spectrum of 2-hydroxy-3-keto-d-glucal.**
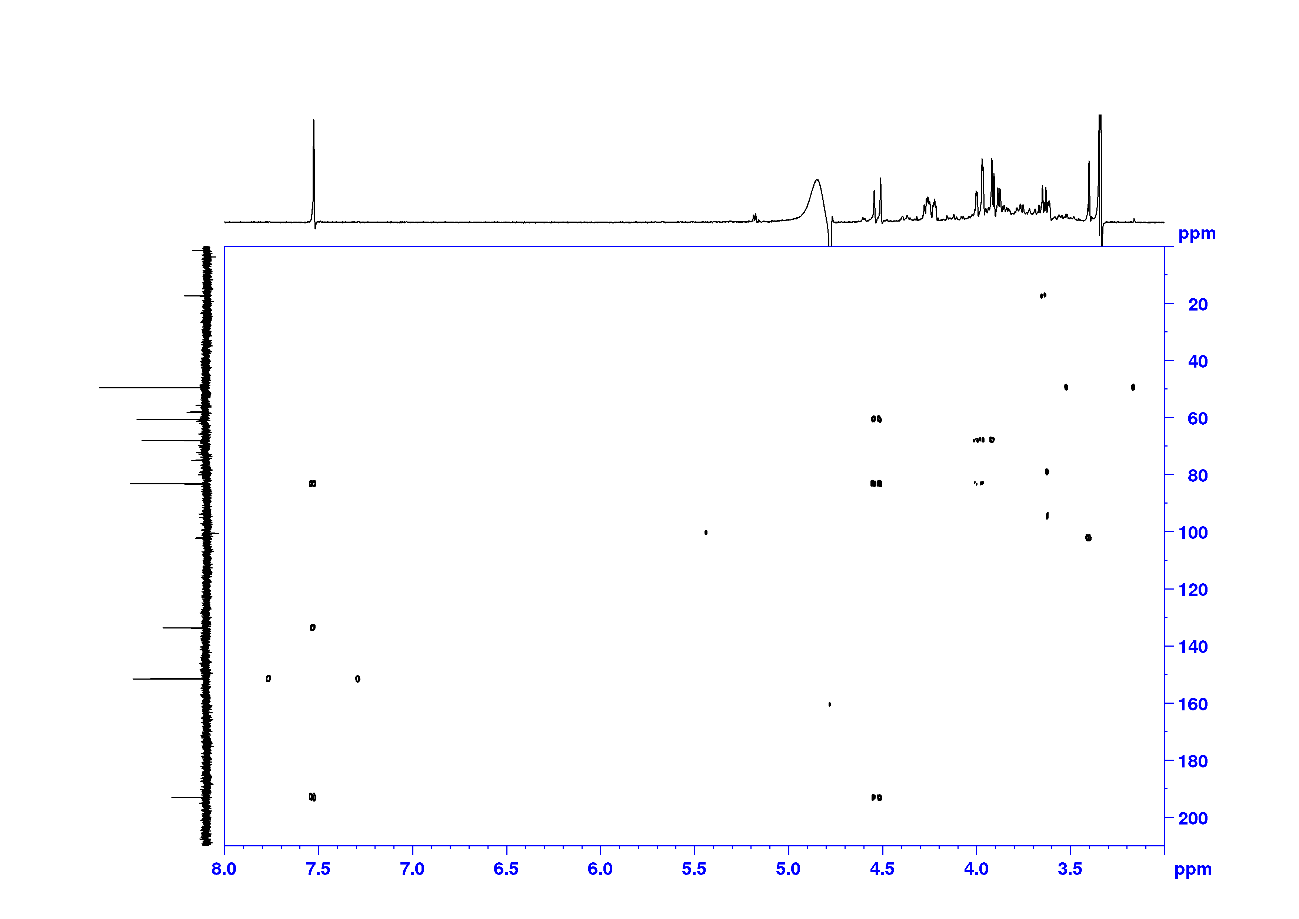


**Fig. S9: HRMS spectrum of 2-hydroxy-3-keto-d-glucal, recorded in positive modality.**


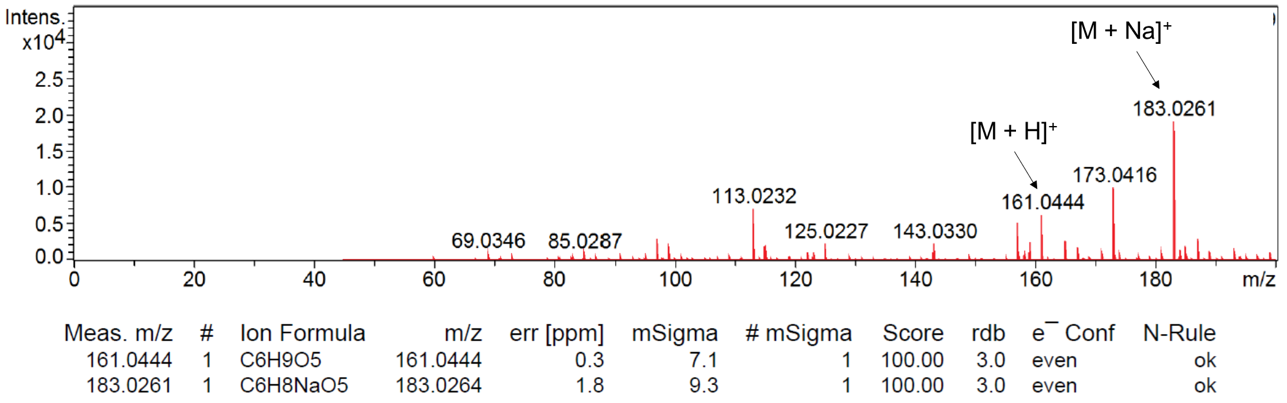

**Fig. S10: HRMS spectrum of 3-keto-d-glucose, recorded in positive modality.**
